# *Toxoplasma* MIC2 Is a Major Determinant of Invasion and Virulence

**DOI:** 10.1371/journal.ppat.0020084

**Published:** 2006-08-18

**Authors:** My-Hang Huynh, Vern B Carruthers

**Affiliations:** W. Harry Feinstone Department of Molecular Microbiology and Immunology, Johns Hopkins Bloomberg School of Public Health, Baltimore, Maryland, United States of America; Stanford University, United States of America

## Abstract

Like its apicomplexan kin, the obligate intracellular protozoan Toxoplasma gondii actively invades mammalian cells and uses a unique form of gliding motility. The recent identification of several transmembrane adhesive complexes, potentially capable of gripping external receptors and the sub-membrane actinomyosin motor, suggests that the parasite has multiple options for host-cell recognition and invasion. To test whether the transmembrane adhesin MIC2, together with its partner protein M2AP, participates in a major invasion pathway, we utilized a conditional expression system to introduce an anhydrotetracycline-responsive *mic2* construct, allowing us to then knockout the endogenous *mic2* gene. Conditional suppression of MIC2 provided the first opportunity to directly determine the role of this protein in infection. Reduced MIC2 expression resulted in mistrafficking of M2AP, markedly defective host-cell attachment and invasion, the loss of helical gliding motility, and the inability to support lethal infection in a murine model of acute toxoplasmosis. Survival of mice infected with MIC2-deficient parasites correlated with lower parasite burden in infected tissues, an attenuated inflammatory immune response, and induction of long-term protective immunity. Our findings demonstrate that the MIC2 protein complex is a major virulence determinant for *Toxoplasma* infection and that MIC2-deficient parasites constitute an effective live-attenuated vaccine for experimental toxoplasmosis.

## Synopsis

Toxoplasma gondii is a protozoan parasite that infects a broad range of hosts including humans. In people with weakened immunity resulting from HIV/AIDS or immune-suppressive treatment following organ transplantation, reactivation of a chronic T. gondii infection represents a serious threat, potentially leading to lethal disease within the brain, heart, or lungs. As an intracellular parasite, invasion into a host cell is a critical first step in ensuring parasite survival during infection. By using a regulatable expression system, this study shows that an adhesive protein called MIC2 is a limiting component of the parasite's invasion system and that it is required for the corkscrew-like movement of the parasite. Moreover, infection of mice with parasites lacking MIC2 no longer resulted in an acute infection leading to death. Not only do mice survive infection, they are protected from infection with a lethal dose of wild-type parasites, indicating an induction of protective immunity. In addition to having implications for the development of live-attenuated vaccines, this work suggests that novel treatment strategies directed at MIC2 may limit the severity of *Toxoplasma* infections.

## Introduction

Apicomplexan parasites cause significant human and animal diseases such as toxoplasmosis *(Toxoplasma gondii),* malaria (Plasmodium spp.), cryptosporidiosis (Cryptosporidium spp.), and coccidiosis (Eimeria spp.). Among the invasion-related apical structures shared by these parasites are the secretory micronemes, which harbor adhesive proteins involved in gliding motility and cell invasion [1–3]. Recent knockout studies have shown that several *Toxoplasma* micronemal proteins (MICs) have significant roles in invasion and virulence; however, no single gene disruption completely abolished infection in vitro or in vivo [4–6]. The transmembrane adhesin MIC2 has long been proposed to play a central role in gliding motility and cell invasion, yet its precise contribution has not been fully established due to lack of strong genetic evidence. Although MIC2 contains several conserved extracellular adhesive domains for receptor-binding and a short cytosolic domain that connects via aldolase to the actinomyosin “glideosome” [7], it remains unclear whether it functionally overlaps with the numerous other transmembrane adhesins that have emerged from the recent sequencing of the *Toxoplasma* genome. Moreover, the recent discovery of other proteins that are more intimately associated with the moving junction raises questions regarding the precise contribution of MIC2 to active entry [8]. Since MIC2 is a member of the conserved thrombospondin-related anonymous protein (TRAP) family of adhesins, it may serve as a valuable model for understanding the function of this family in active cell invasion by other apicomplexan parasites.

To address the role of MIC2 in *T. gondii,* we generated a conditional knockout mutant using a tetracycline-regulatable system [9]. We show that MIC2-deficient parasites are severely defected in their ability to attach to and invade host cells and are transformed to a non-lethal strain in the mouse model of infection.

## Results/Discussion

### Reduced MIC2 Expression Severely Compromises Attachment and Invasion

**Figure 1:**
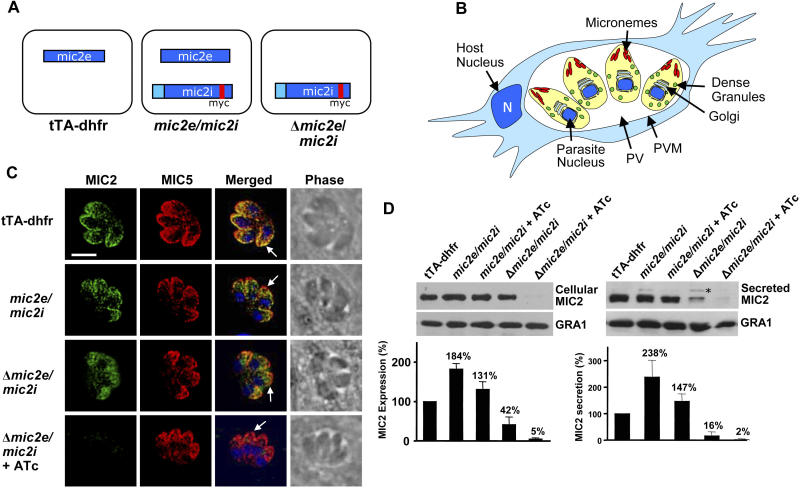
Conditional Expression of MIC2 (A) Schematic diagram showing the strains in the study, including the parental strain (tTA-dhfr), and the *mic2e/mic2i* strain with both endogenous *(mic2e)* and induced *(mic2i)* copies of mic2. A knockout of *mic2e* leaves the regulatable *mic2i* in *Δmic2e/mic2i.* (B) Illustration of intracellular structures including the micronemes, dense granules, Golgi, PV, PV membrane, and host and parasite nuclei in a four-parasite vacuole. (C) Expression and localization of MIC2 (green) in intracellular parasites with another micronemal marker MIC5 (red). Arrows indicate the apical pole of parasites. Scale bar, 5 μm. (D) Western blot analyses showing MIC2 steady-state expression (left blot) and secretion (right blot) levels. Asterisk indicates the full-length myc-tagged MIC2, which is secreted in *mic2e/mic2i* and Δ*mic2e/mic2i* parasites based on probing with a myc antibody (unpublished data). Blots were probed with anti-MIC2 6D10 (top blots) and mouse anti-GRA1 (bottom blots), a dense granule protein, to normalize loading in all lanes. The bar graphs represent the relative percentages of MIC2 expressed in each strain and treatment compared to the reference tTA-dhfr level (100%) quantified from direct chemiluminescent imaging. Results are mean ± s.e.m, *n* = 3.

Since previous attempts to directly disrupt the *mic2* gene have been unsuccessful, a conditional knockout system was employed. This scheme utilizes a transcriptional transactivator (tTA) protein that regulates expression of genes containing tetracycline operator cassettes upstream of the transcription start site. Genes are constitutively transcribed until the addition of tetracycline releases the tTA from the operator cassettes, preventing further transcription. The parental strain was generated by introducing the tTA into the RH type I strain [9]. The “reference” strain in this study, tTA-dhfr, expresses tTA and a dihydrofolate reductase-thymidylate synthase (dhfr-ts) to control for expression of this selectable marker. Strains generated in this study are shown schematically in Figure 1A. The tetracycline-responsive *mic2,* henceforth referred to as *mic2i,* was transfected into the tTA strain using the dhfr-ts marker and an individual clone, named *mic2e/mic2i* to indicate the presence of both the endogenous and inducible *mic2* genes, was isolated by pyrimethamine selection. A knockout plasmid containing the 5′ and 3′ *mic2* genomic flanking regions and the full-length chloramphenicol acetyl transferase (CAT) selectable marker was then used to replace the endogenous *mic2* by homologous recombination in the *mic2e/mic2i* clone; a resulting “knockout” clone, Δ*mic2e/mic2i,* was isolated by chloramphenicol selection. The presence of the inducible *mic2* or absence of the endogenous *mic2* was confirmed by PCR and Southern blotting (Figure S1).

To determine the localization of *mic2e* and *mic2i* in all strains, immunofluorescence staining of intracellular parasites was performed. Figure 1B illustrates the spatial distribution of structures and organelles in a four-parasite vacuole inside a host cell. Figure 1C shows dual-immunostaining of MIC2 and another microneme protein, MIC5. In the tTA-dhfr strain, as observed with wild-type parasites (unpublished data), both proteins occupied the apical micronemes (Figure 1C, top row). Similarly, the *mic2e/mic2i* strain showed a large portion of MIC2 co-localizing with MIC5 (Figure 1C, second row). In Δ*mic2e/mic2i* parasites, which only express the inducible copy, MIC2 was present both in the micronemes and in vesicles positioned centrally within the anterior region (Figure 1C, third row). This incomplete trafficking to the micronemes is likely a consequence of using a heterologous promoter, as seen in other studies [5,10]. When anhydrotetracycline (ATc), a non-cytotoxic analog of tetracycline, was added to Δ*mic2e/mic2i* parasites, there was a notable reduction in MIC2 expression (Figure 1C, bottom row). When levels of MIC2 in cell lysates or in the excreted-secreted antigen fraction are examined by quantitative immunoblotting (Figure 1D), the recombinant strains exhibited incremental changes in MIC2 expression and secretion ranging from 184% and 238%, respectively, in *mic2e/mic2i* without ATc to only 5% and 2%, respectively, in Δ*mic2e/mic2i* +ATc. All values are expressed relative to tTA-dhfr (100%).

**Figure 2:**
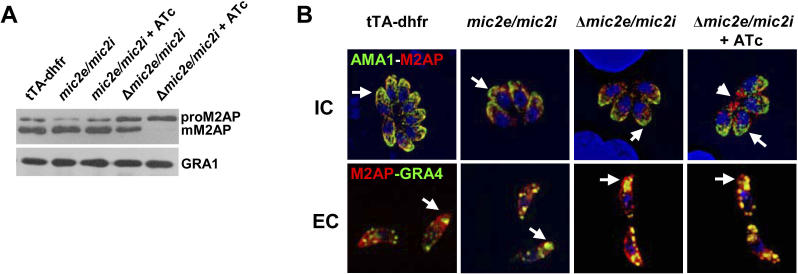
Proteolytic Maturation and Localization of M2AP Is Dependent on Expression of MIC2 (A) Western blot of whole cell lysates showing steady-state expression levels. Blots were probed as indicated. Proform M2AP (proM2AP); mature M2AP (mM2AP). (B) Expression and localization of M2AP in intracellular (IC) and extracellular (EC) parasites. Top panels: dual staining of M2AP (red) with AMA1 (green) in IC parasites showing mislocalization of M2AP in the PV of Δ*mic2e/mic2i* + ATc parasites. Bottom panels: immunostaining of M2AP (red) and GRA4 (green) in EC parasites shows a progressive increase in M2AP expression in dense granules, particularly in Δ*mic2e/mic2i* + ATc parasites. Arrows indicate the apical poles of parasites and an arrowhead indicates the PV.

Previous studies have shown that MIC2 and its partner protein M2AP are intimately associated soon after synthesis [11], and that the absence of M2AP causes mislocalization and reduced secretion of MIC2 [4]. Similarly, we found that under-expression of MIC2 abolished the proteolytic maturation of M2AP from its proform to mature form and led to its inappropriate targeting to dense granules and the parasitophorous vacuole (PV). More unprocessed proform of M2AP was observed in the lysates of Δ*mic2e/mic2i* compared to tTA-dhfr and *mic2e/mic2i,* and upon ATc treatment, almost all M2AP was found in the uncleaved form (Figure 2A). In intracellular tTA-dhfr and *mic2e/mic2i* parasites, M2AP largely showed normal co-localization with another micronemal marker, AMA1, in the apical pole (Figure 2B, top panels). Δ*mic2e/mic2i* parasites, which express 42% MIC2, exhibited a fraction of M2AP associated with dense granules and the PV. In Δ*mic2e/mic2i* +ATc parasites (5% MIC2), there was almost no co-localization of M2AP with AMA1 in the micronemes and staining was observed mostly in the dense granules and the PV. In extracellular parasites, almost all M2AP co-localized with GRA4 in the dense granules of Δ*mic2e/mic2i* +ATc parasites (Figure 2B, bottom panels). Secretion via the dense granules constitutes the default pathway and without its partner, unprocessed proM2AP is diverted from the micronemal route to the dense granules. These findings confirm that both members of the complex are required for correct micronemal targeting. Given the incomplete trafficking of the inducible MIC2, we examined other microneme proteins to determine whether proper trafficking, expression, and processing was affected. As shown in Figures 1C and 2B, MIC5 and AMA1 were targeted correctly to the micronemes. By Western blot analysis, MIC3, MIC4, MIC6, MIC10, and AMA1 were expressed and processed at the normal levels in Δ*mic2e/mic2i* with or without ATc (unpublished data). Therefore, we conclude that depletion of the MIC2 complex does not widely affect expression and trafficking of other microneme proteins.

**Figure 3:**
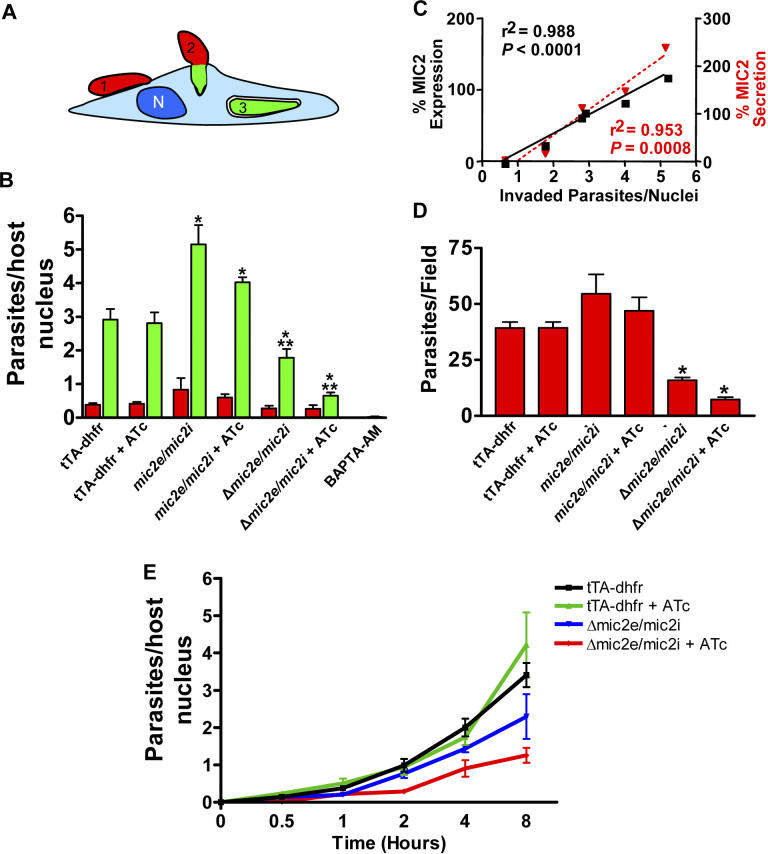
Invasion Phenotypes Associated with Reduced MIC2 Expression (A**)** Illustration of the red-green invasion assay based on differential immunolabeling. Invading parasites (step 2) were counted as green. (B) Quantification of the red-green invasion assay: red bars, attached extracellular parasites; green bars, invading and invaded parasites. A single asterisk indicates a statistically significant difference compared to tTA-dhfr; double asterisk indicates statistical difference compared to *mic2e/mic2i* + ATc (two-tailed Student's *t*-test). BAPTA-AM-treated parasites were included as a positive control for an attachment/invasion defect. Data are mean values ± s.e.m. of four separate experiments, each with three replicates and counting eight randomly selected fields per well. (C) Correlation and linear regression of the percentage of MIC2 expression in cell lysates (left Y-axis and black line) and the percentage of MIC2 secretion (right Y-axis and red dashed line) with the numbers of invaded parasites. (D**)** Attachment to glutaraldehyde-fixed host cells. An asterisk indicates that attachment was significantly lower than tTA-dhfr (*p* < 0.002, two-tailed Student's *t*-test). Data were compiled from three separate experiments, counting six fields per well per clone. (E) Time-course invasion of tTA-dhfr and Δ*mic2e/mic2i* parasites ± ATc over an 8 h period. Data represent five individual experiments with three replicates within each experiment.

We next used a red-green differential antibody staining assay to determine the role of the MIC2 complex in parasite attachment and invasion [4,12]. As depicted in Figure 3A, extracellular parasites are labeled red while intracellular parasites are labeled green. Invasion by the *mic2e/mic2i* strain without or with ATc treatment was significantly higher (*p* = 0.0138 and 0.0188, respectively) than tTA-dhfr, a finding that is consistent with the over-expression and over-secretion of MIC2 in this strain (Figure 1D bar graph and Figure 3B). Δ*mic2e/mic2i,* which only secretes MIC2 at 16% of normal levels, showed a ~60% invasion efficiency versus tTA-dhfr parasites (*p* = 0.0325). This defect was further magnified by addition of ATc when MIC2 secretion dropped to 2% and invasion was limited to only ~20% (*p* = 0.0005). The residual invasion levels observed are likely due to the small amount of MIC2 remaining after ATc treatment as well as the presence of other adhesive MIC complexes. MIC2 expression levels in cell lysates closely correlate (Figure 3C) with numbers of invaded parasites (two-tailed Pearson test: *r* ^2^ = 0.988, *p* < 0.0001; Spearman test: *p* = 0.0028), as does MIC2 secretion (Pearson: *r* ^2^ = 0.953, *p* = 0.0008, Spearman: *p* = 0.0167) with invaded parasites. We observed a similar pattern (Figure 3D) using glutaraldehyde-fixed host cells, which are receptive to parasite attachment but refractory to invasion [4,13]. Therefore, the entry defect is principally due to an inability to adhere properly to the target cell, although we cannot rule out an additional defect in the penetration step that follows attachment. To determine whether the invasion phenotype was time-dependent, we examined invasion of tTA-dhfr and Δ*mic2e/mic2i* with and without ATc over the course of 8 h. Although the difference between parental and MIC2-deficient strains was less pronounced at later time points, MIC2-deficient parasites remained at least 40%–50% less proficient in invasion (Figure 3E). Collectively, our findings strongly suggest that the MIC2 complex is a major determinant of *Toxoplasma* adhesion and invasion.

### MIC2 Does Not Influence Intracellular Replication or Egress, but Contributes to Helical Gliding

To examine whether MIC2 expression influences other events in the parasite's life cycle, we assessed the MIC2-regulated strains for replication, egress, and gliding motility. For replication, vacuoles were enumerated after 17 h and 26 h of growth, and scored for the number of vacuoles with 1, 2, 4, 8, and 16 parasites per vacuole. No significant difference between tTA-dhfr and Δ*mic2e/mic2i* + ATc-treated parasites was observed (Figure S2), confirming that MIC2 does not influence replication. After intracellular replication, parasites exit host cells in an active process that involves the activation of calcium signaling within the parasite [14]. As measured by timed video microscopy after inducing egress by treatment with calcium ionophore [15] or DTT [16], there was no detectable defect in the ability of the Δ*mic2e/mic2i* + ATc parasites to exit host cells (Table 1 and Videos S1 and S2). A similar specialized function in invasion, but not egress, was recently found for *Toxoplasma* AMA1 [6,8] implying that the parasite uses distinct molecules for entry and exit.

**Table 1:**
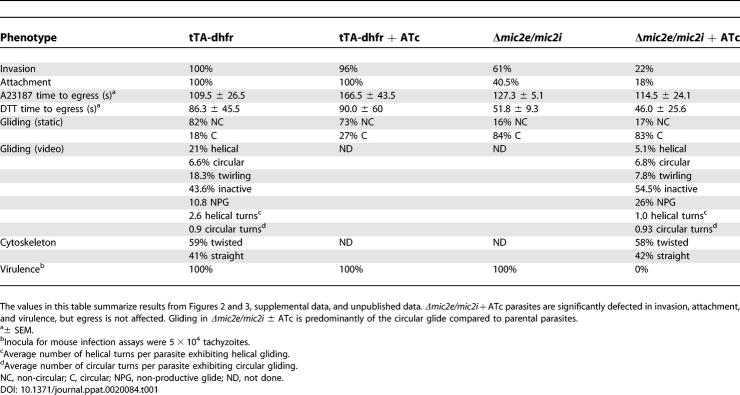
Summary of Phenotypes Associated with MIC2-Depleted Parasites

**Figure 4:**
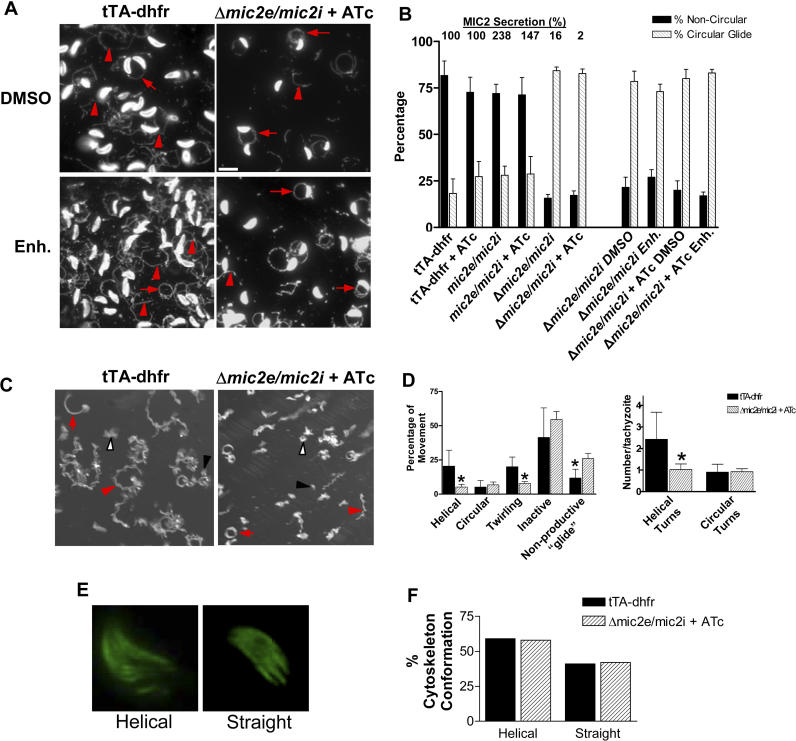
Gliding Phenotypes by Static Assay and Live Video Microscopy (A) Assessment of gliding motility by trail deposition. Top panels: DMSO is used as a solvent control. Scale bar, 15 μm. Bottom panel: UVT153753 (Enh) enhancer-treated parasites. Arrowheads indicate non-circular trails and arrows denote circular trails. (B) Quantification of non-circular and circular trails is presented on the left half of the graph, UVT153753- or DMSO-treated Δ*mic2e/mic2i* ± ATc parasites are represented on the right half. Results are mean ± s.e.m of at least three experiments. Black bars, % non-circular gliding; grey hatched bars, % circular glide. (C) Maximum projection images created from frames 1–60 (1 min videos taken at 1 frame per s). Red arrows, circular glide; red arrowheads, helical glide; closed black arrowhead, non-productive “gliding” parasite; open black arrowhead, twirling parasite. (D) Quantification of types of movement in live gliding parasites; error bars represent standard deviation. An asterisk indicates a statistically significant difference between tTA-dhfr and Δ*mic2e/mic2i*+ ATc parasites (*p* < 0.02). (E) Immunofluorescent images of anti-tubulin-stained tachyzoites showing helical and straight cytoskeletons. (F) Bar graph represents enumeration of at least 85 tachyzoite cytoskeletons. Enumeration was performed in a blinded fashion.

Gliding motility plays a central role in parasite migration, invasion, and egress (reviewed in [17]). *Toxoplasma* motility has been observed in three forms: helical, circular, and twirling movements. Helical gliding is thought to be the main form that precedes invasion of host cells [18], and this mode of gliding deposits long trails that are wide-arcing or straight (non-circular). A typical field of trails left by wild-type and tTA-dhfr parasites shows both non-circular and circular trails (Figure 4A, top left panel). Δ*mic2e/mic2i* parasites do not adhere as well to the slides and therefore leave fewer trails, but the ones that are left are distinctly of the circular glide, with few long trails (Figure 4A, top right panel). Many of the Δ*mic2e/mic2i* circular trails are comprised of several trails tightly wound together, indicating that each one is created by the gliding activity of a single parasite performing multiple laps. Enumeration of the types of trails showed a marked loss of non-circular gliding in Δ*mic2e/mic2i* and Δ*mic2e/mic2i* + ATc compared to tTA-dhfr (*p* = 0.0002 and *p* = 0.0003, respectively; Figure 4B). Interestingly, this change in gliding pattern was seen with Δ*mic2e/mic2i* regardless of whether parasites were treated with ATc. This could be because untreated Δ*mic2e/mic2i* parasites secrete MIC2 at only 16% of normal levels, an amount that is apparently below the threshold for sustaining non-circular motility. Although *mic2e/mic2i* parasites express greater levels of MIC2, the percentage of non-circular versus circular trails left were similar to those of tTA-dhfr (Figure 4B), indicating that over-expression of MIC2 does not increase non-circular gliding. Assays were then performed using an enhancer of gliding and invasion (UVT153753) identified in a recent small molecule screen [19] to examine whether this would restore non-circular gliding in Δ*mic2e/mic2i* parasites. Although more parasites were activated, resulting in greater trail deposition, the proportion of non-circular versus circular trails remained the same (Figure 4A, bottom panels and 4B).

Parasite gliding was also examined by live video microscopy. The movement of individual parasites is displayed as maximum projections of video frames 1–60 (Figure 4C), in which all forms of gliding were illustrated. Gliding modalities were enumerated by viewing eight independent videos of each strain. This analysis confirmed the above findings. Δ*mic2e/mic2i* + ATc parasites exhibited normal circular motility but less helical gliding, and they completed fewer helical movements per tachyzoite compared to tTA-dhfr parasites (Figure 4D, Table 1, and Videos S3 and S4). The video analysis further showed that Δ*mic2e/mic2i* + ATc parasites are defective in the twirling motion, in which the parasite pivots on its posterior end yielding a star-like pattern on the maximum projection images. Moreover, a greater proportion of Δ*mic2e/mic2i* + ATc parasites were either inactive or exhibited a non-productive glide. For this latter movement the parasite seems to initiate a movement, but fails in achieving a helical, circular, or twirling action.

Because the helical gliding motion is thought to be dependent upon the semi-helical nature of the parasite's tubulin-based cytoskeleton, we examined whether a change in the cytoskeletal pattern (helical/twisted versus straight; Figure 4E) might be responsible for the loss of helical gliding in Δ*mic2e/mic2i* + ATc parasites. Equal numbers of parasites with helical or straight cytoskeletons were observed in tTA-dhfr and in Δ*mic2e/mic2i* + ATc (Figure 4F), indicating that there was no change in this structure that could account for the large decrease in helical gliding. Alternatively, we propose that Δ*mic2e/mic2i* parasites are defective in helical gliding because this motion requires the parasite to raise its apical end and twist up from the substrate. This upward motion probably requires maximum traction and adhesion at the parasite's posterior end to counteract gravitational forces and elevate the apical pole. Since a similar upward thrust is necessary to initiate twirling, we suggest that MIC2 is needed to provide sufficient substrate binding to produce both helical and twirling motility. Conversely, circularly gliding parasites remain parallel to the substrate, and therefore they are still able to perform this motion in the near absence of the MIC2 complex. Thus, unlike *Plasmodium* sporozoites, which are entirely dependent on the thrombospondin-related anonymous protein family of adhesins (TRAP) expression for all modes of gliding [20], *Toxoplasma* appears to only require the MIC2 complex for helical gliding and twirling motility; other microneme-derived adhesive molecules are presumably sufficient for circular gliding in the absence of MIC2.

### MIC2-Deficient Parasites Are Avirulent

**Figure 5:**
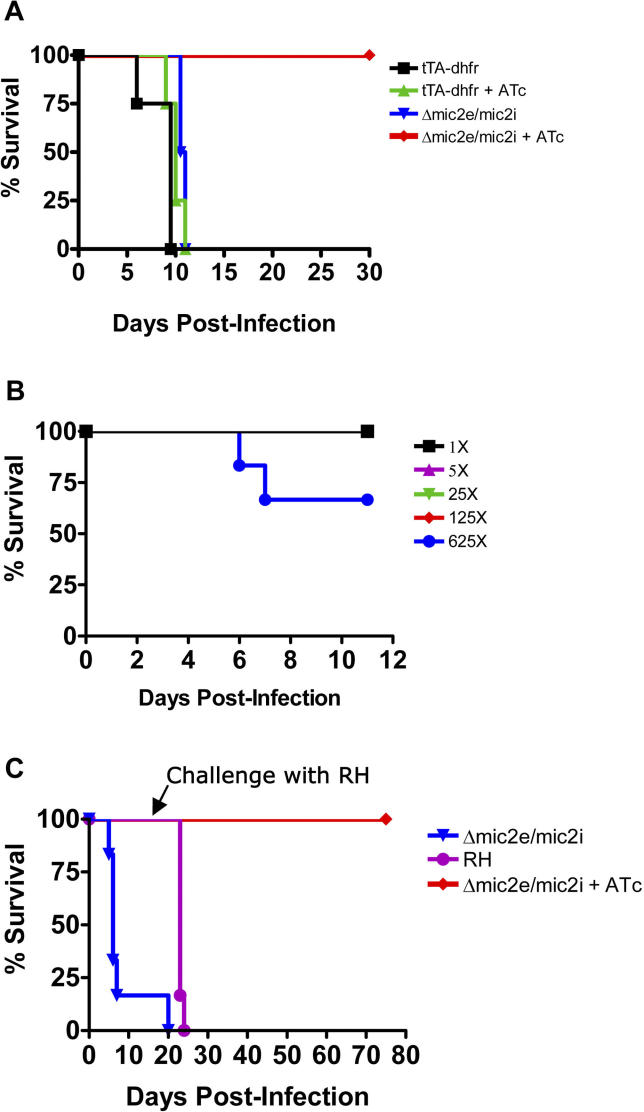
MIC2-Depleted Parasites Are Avirulent in Mice and Confer Protective Immunity to Reinfection (A) 5 × 10^4^ tachyzoites of tTA-dhfr or Δ*mic2e/mic2i* ± ATc were intraperitoneally injected into four BALB/c mice in each group. (B) 5-fold increases in infection dosage with Δ*mic2e/mic2i* +ATc; six mice were infected in each group. (C) Six mice infected with Δ*mic2e/mic2i* + ATc were challenged with 150 tachyzoites of RH at day 16 post-infection. A group of control mice were infected with RH at day 16.

To examine the role of the MIC2 complex in *Toxoplasma* infection, mice were infected with tTA-dhfr or Δ*mic2e/mic2i* ± ATc parasites, and MIC2 expression levels were suppressed by providing ATc in the drinking water. The type I RH strain, the original parent of the clones used in this study, is extremely virulent with a lethal dose of fewer than five parasites [21]; however, in our hands both tTA and tTA-dhfr require higher doses (5 × 10^4^ tachyzoites) to achieve normal time-till-death kinetics. While the basis of this partial attenuation remains unknown, we believe that tTA-dhfr is still a valuable reference strain for examining the role of MIC2 in lethal infection. As expected [9], administration of ATc had no effect on tTA-dhfr infection and all mice died by day 11 (Figure 5A). Similarly, all mice infected with Δ*mic2e/mic2i* minus ATc died by day 11, suggesting that moderate levels of MIC2 are still sufficient to support lethal infection. As shown above, these parasites fail to produce long, non-circular trails, yet they show the same virulence level as tTA-dhfr parasites. This implies that long distance migration is not strictly required for virulence, as suggested by a correlation of this property with naturally occurring virulent strains [22]. Strikingly, mice inoculated with Δ*mic2e/mic2i* + ATc all survived the acute infection, even when followed for up to 5 mo post-infection. To further assess this virulence defect, mice were infected with 5-fold increasing doses of Δ*mic2e/mic2i* + ATc. All mice survived inoculation with 5 × 10^4^, 2.5 × 10^5^, 1.25 × 10^6^, and 6.25 × 10^6^ parasites, and two-thirds of the mice also survived infection with 3 × 10^7^ parasites, i.e., 625X the lethal dose for tTA-dhfr or Δ*mic2e/mic2i* minus ATc (Figure 5B). Seroconversion of all surviving mice was confirmed by ELISA. Three groups of mice were infected in another experiment to determine whether surviving mice were protected from subsequent infection (Figure 5C). The first group was infected at day 0 with Δ*mic2e/mic2i* minus ATc and again succumbed to the infection. A second group infected with Δ*mic2e/mic2i* + ATc again survived the acute infection and survived a challenge infection with 150 RH tachyzoites administered at day 16, whereas a third group of naïve mice infected with the same dose of RH tachyzoites died within 10 d of the challenge. Δ*mic2e/mic2i* + ATc-infected mice also survived a similar challenge with RH parasites 60 d post-infection (unpublished data), indicating that Δ*mic2e/mic2i* + ATc parasites induce long-term protective immunity. Collectively, these results demonstrate that expression of the MIC2 complex is required for *Toxoplasma* lethal infection and MIC2-deficient parasites constitute an effective live-attenuated vaccine.

**Figure 6:**
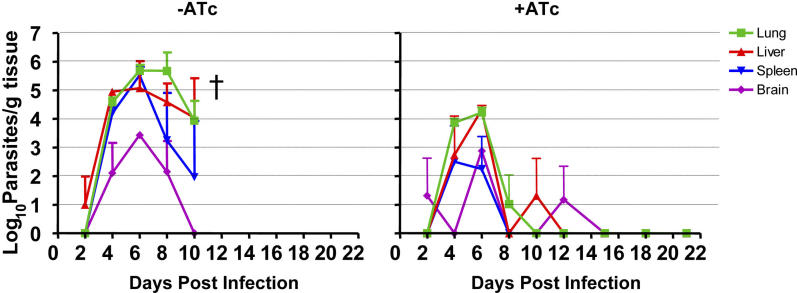
Δ*mic2e/mic2i* + ATc Parasites Fail to Reach High Tissue Levels and Are Cleared Parasite tissue burden of mice during infection with Δ*mic2e/mic2i* treated with and without ATc. Organs from three mice were isolated at each time point and analyzed by rtqPCR using parasite specific primers. The normal time-till-death Δ*mic2e/mic2i* without ATc is indicated by a “†.”

To distinguish whether the virulence defect was due to an inability to establish, disseminate, or sustain the infection, we monitored parasite burden in various tissues by real-time quantitative PCR (rtqPCR). In mice infected with Δ*mic2e/mic2i,* peak parasitemia occurred at day 6 post-infection (Figure 6; note: log_10_ scale). The infection partially waned at day 8, consistent with the onset of the adaptive immune response, which nonetheless is insufficient to prevent a lethal outcome. A similar peak at day 6 was observed with Δ*mic2e/mic2i* + ATc parasites, but the levels of parasitemia were several logs lower in most organs compared to Δ*mic2e/mic2i*-infected mice. For example, parasite burden in the lung of Δ*mic2e/mic2i* + ATc-infected mice at day 6 was ~100-fold lower than in Δ*mic2e/mic2i*-infected mice. The parasite burden in the spleen was ~1,000-fold lower in Δ*mic2e/mic2i* + ATc-infected mice, perhaps due to more effective phagocytic clearance in this secondary lymphoid organ. Parasite tissue burden declined to below detection limits of rtqPCR for all tissues by day 15 post-infection implying parasite clearance. No encysted or free parasites were seen in brain homogenates from the surviving mice. Also, naïve mice inoculated with brain homogenates from surviving mice did not seroconvert, further supporting parasite clearance. We conclude that the MIC2 complex is not required to establish or disseminate the infection but is necessary to rapidly expand and sustain the infection within tissues.

**Figure 7:**
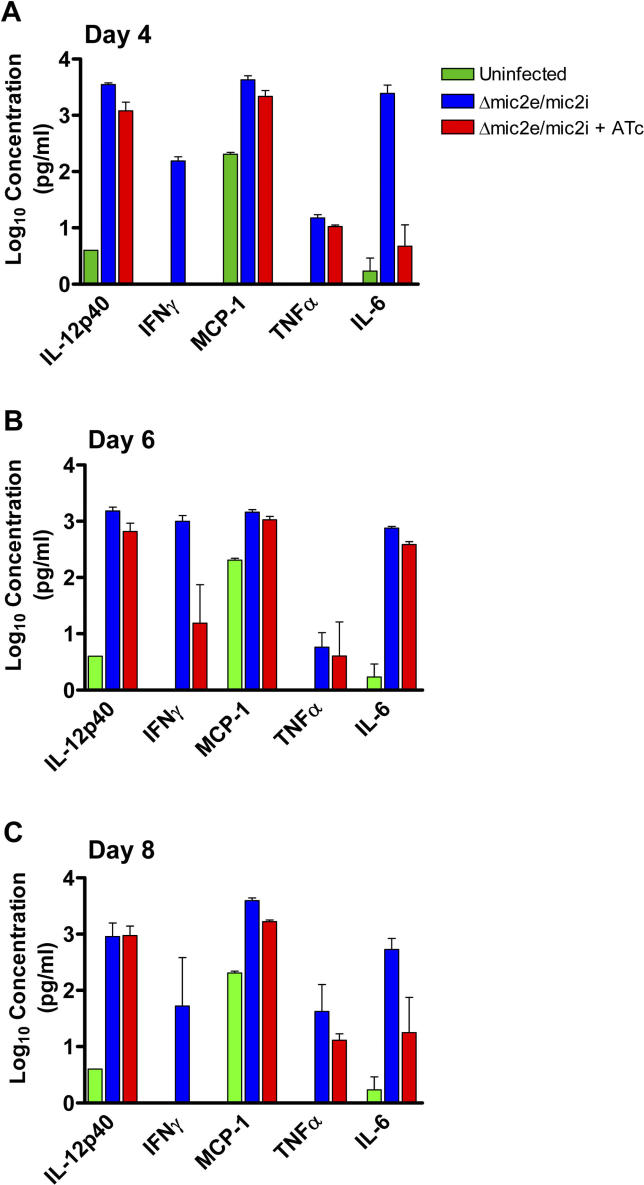
Mice Infected with MIC2-Deficient Parasites Produce Lower Levels of Inflammatory Cytokines Mice were infected with Δ*mic2e/mic2i* ± ATc and treated with or without ATc in drinking water. Serum was collected from three mice on days 4 (A), 6 (B), and 8 (C) post-infection. Cytokine levels were determined by a quantitative cytokine protein microarray analysis.

The overproduction of pro-inflammatory cytokines—particularly interleukin-12 (IL-12), interferon-gamma (IFNγ), and tumor necrosis factor alpha (TNFα)—by mice infected with virulent strains of T. gondii is thought to contribute to death due to overt apoptosis in vital organs [23]. To investigate the basis of mouse survival during infection with Δ*mic2e/mic2i* + ATc, we tested pro-inflammatory cytokine levels at day 4, 6, and 8 post-infection. Moderately lower levels of IL-12p40, TNFα, and monocyte chemoattractant protein-1(MCP-1) were seen in Δ*mic2e/mic2i* +ATc mice compared to Δ*mic2e/mic2i*-infected mice (Figure 7; note: log_10_ scale). Interestingly, IFNγ was below the level of detection on day 4 (Figure 7A), was ~100-fold lower than Δ*mic2e/mic2i* without ATc on day 6 (Figure 7B), and again dropped to below detection levels on day 8 (Figure 7C). On day 4, mice infected with Δ*mic2e/mic2i* +ATc also showed substantially lower levels of IL-6, an inducer of T- and B-cell growth and differentiation, compared to mice infected with Δ*mic2e/mic2i* without ATc treatment. Therefore, infection results in an immunologic response that is sufficient to clear the infection without causing lethal inflammatory disease.

Disruption of other T. gondii genes has previously been shown to attenuate parasite virulence. Fox and Bzik demonstrated that de novo pyrimidine biosynthesis is essential in vivo, and parasites are incapable of scavenging sufficient amounts of pyrimidines from the host cell to support intracellular growth. In the mouse model, these uracil auxotrophs are highly attenuated, and infected mice were also protected from challenge infection with a virulent strain [24]. Another study involving a double-knockout of the micronemal proteins MIC1 and MIC3 led to a similar attenuation of virulence and a reduction in brain cysts upon challenge with a non-virulent, cyst-forming T. gondii strain [5]. Our current study with a MIC2-deficient strain expands on the in vivo consequences of infection by providing additional data on parasite burden in various tissues and the inflammatory cytokine response mounted by the host. Our findings indicate that the lower organ burden of Δ*mic2e/mic2i* + ATc-infected mice produces a less pronounced inflammatory response leading to host survival and long-term adaptive immunity to re-infection. Interestingly, similar differences in parasite tissue burden and inflammatory response were seen between virulent type I and avirulent type II strains [23,25], indicating that disruption of the single *MIC2* locus effected a major virulence conversion reminiscent of differences seen in naturally occurring strains. Because of the transient infection they produce, Δ*mic2e/mic2i* parasites may be a valuable experimental vaccine for understanding innate and adaptive immune mechanisms leading to protective immunity from toxoplasmosis.

## Materials and Methods

### Plasmid constructs.

The construct for the inducible copy of the *MIC2* gene was generated by PCR amplification of the pHLEM TgMIC2.HA9 (from L. D. Sibley) (containing a myc tag at position 2034 of the MIC2 cDNA) with forward primer pTetOS1mycGFPMIC2.F (5′ gatcgaattccctttttc**gacaaa**ATGAGACTCCAACGCGAG) and reverse primer TgMIC2.2310.PacI.R (5′ gatcaattaattCTACTCCATCCACATATC) to incorporate an *Eco*RI restriction site (underlined) and a short T. gondii-specific sequence (in bold) upstream of the initiator codon and a *Pac*I restriction site downstream of the last codon. The vector pT7SAG1mycGFP was digested with *Eco*RI and *Pac*I to remove the GFP cassette, and the myc-tagged MIC2 PCR product was ligated in, resulting in the vector pT7SAG1mycMIC2. The construct (mic2CATmic2) for precise (ATG to TAA) replacement of the TgMIC2 gene was generated by insertion of 1,446 bps of 5′ and 1,183 bps of 3′ flanking sequences of TgMIC2 in a selectable vector containing the cDNA coding for CAT controlled by the tubulin promoter.

### Parasites, transfection, and selection.

T. gondii tachyzoites were maintained by growth on monolayers of human foreskin fibroblasts (HFF) in Dulbecco's Modified Eagles Medium (DMEM, GIBCO, San Diego, California, United States) containing 10% Fetal Bovine Serum (GIBCO), 2 mM Glutamine, 10 mM HEPES, 50 μg/ml Penicillin/Streptomycin (D10 Complete). The tTA parasite clone (expressing the tet-transactivator) was generated as described in Meissner et al. [9] and maintained in HFF cells without selection. To generate stable transformants expressing the endogenous and inducible copies of MIC2, 5 × 10^7^ extracellular tTA tachyzoites were co-transfected with 100 μg of pT7SAG1mycMIC2 and 5 μg of the pDHFR-TSc3/M2M3 plasmid [26] conferring pyrimethamine resistance. A single parasite clone was obtained by selection with pyrimethamine 1 d after transfection followed by limiting dilution. To knockout the endogenous copy of MIC2, the *mic2e/mic2i* clone was transfected as described above with 30 μg of mic2CATmic2, and transfected pools of parasites were PCR-tested for the presence of a knockout before selection with chloramphenicol and cloned by limiting selection.

### PCR primers.

Primers to detect the endogenous *MIC2* gene were directed at an intron and the 3′ flanking region: forward primer TgMIC2.251794.F (5′ GAGACACAGCAAGCCCAGAAG) and reverse primer TgMIC2.253320.R (5′ GGCACACACCAAGCCATCAGGA). Primers used to determine the proper insertion of the CAT selectable marker in the *MIC2* locus were as follows: forward primer CAT.500.F to the 3′ end of CAT (5′ TTCTTCGCCCCCGTTTTCACCA) and reverse primer TgMIC2.253608.R to flanking genomic DNA outside the mic2 locus (5′ GAGACGGCACAAACAAACTG). To determine that the endogenous mic2 had not excised and reintegrated elsewhere, primers against an intron and the 3′ end of the gene were used: forward primer TgMIC2.251794.F (5′ GAGACACAGCAAGCCCAGAAG) and reverse primer TgMIC2.2310.R (5′ GTTCGCGGCCGCTACTCCATCCACATATCAC). To identify clones that had integrated copies of the inducible copy of MIC2 in the genome, primers to the internal MIC2 cDNA and to the myc tag were used: forward primer TgMIC2.800.F (5′ GATCCCCCCAGGATGCCATTTGC) and reverse primer mycTag.R (5′ TTCTGAGATAAGCTTCTGCTC).

### Quantitative Western blotting.

T. gondii cell lysates were run on 10% SDS-PAGE gels and Western blotted with mouse anti-MIC2 (6D10), mouse anti-GRA1, and rabbit anti-M2AP. After adding Supersignal substrate (Pierce Biotechnology, Rockford, Illinois, United States), direct chemiluminescence was captured on a Fuji Film (Tokyo, Japan) LAS-1000 CCD camera system and analyzed using the Fuji Film Image Gauge software. Standard curves were generated for MIC2 and GRA1 separately by measuring the relative arbitrary units of the bands in lanes containing 100%, 75%, 50%, 25%, 12.5%, and 6% of control cell lysates (tTA-dhfr). The GRA1 standard curve was used to equalize loading in each lane, and the amount of MIC2 in the *mic2e*/*mic2i* and Δ*mic2e/mic2i* ± ATc strains were extrapolated from the MIC2 curve after normalization with GRA1.

### Secretion assays.

Secretion assays were performed as previously described [4] with the following modifications: 100 μl of filter-purified tachyzoites (2 × 10^8^/ml in invasion medium (IM): DMEM/20 mM HEPES/3% FBS) was added to 100 μl prewarmed IM in an eppendorf tube and incubated for 20 min at 37 °C. Secretion was arrested by placing the tubes on ice for 5 min and culture supernatants were collected after removing parasites by centrifugation (1,000 *g*, 3 min, 4 °C, twice).

### Indirect immunofluorescence microscopy.

All manipulations were carried out at room temperature. Tachyzoite-infected HFF cells on 8-well chamber slides were fixed with 4% paraformaldehyde-0.02% glutaraldehyde for 20 min, followed by washes in PBS. Fixed cells were permeabilized with 0.1% Triton X-100 in PBS for 15 min, washed and blocked in 10% FBS in PBS for 30 min. The wells were then stained with the primary antibodies followed by Alexa 594 or Alexa 488-conjugated goat anti-mouse or goat anti-rabbit antibodies (Molecular Probes, Eugene, Oregon, United States). After washes in PBS/1%FBS/1%NGS, slides were mounted in Mowiol and fluorescent images were collected at 100X with a Nikon Eclipse E800 (Tokyo, Japan) microscope equipped with an RT Spot Slider CCD camera. Images were deconvolved using the Simple PCI software and assembled using Adobe Photoshop.

### Invasion and attachment assays.

Assays were performed as previously described [4]. For the time course invasion, 2.5 × 10^5^ parasites were loaded in each well of an eight-well chamber slide and incubated for 0.5, 1, 2, 4, and 8 h at 37 °C before fixation. Slides were differentially stained with anti-SAG1 antibodies as per the red-green invasion assay.

### In vitro gliding assay.

Eight-well chamber slides were coated either overnight at 4 °C or 1 h at 37 °C with 50% FBS/50% PBS, and washed with PBS before use. Freshly lysed parasites were syringed, filtered, chased with HHE (Hank's Balanced Salt Solution, 10 mM HEPES, 1 mM EGTA) and centrifuged at 1,000 rpm for 10 min RT. The parasite pellet was resuspended in 5 ml HHE and 500 μl was inoculated into each chamber of the coated slide. The slide was then incubated at 37 °C for 15 min, rinsed in PBS, and fixed in 4% paraformaldehyde/0.02% glutaraldehyde in PBS. Trails left by gliding parasites were visualized by staining with a rabbit anti-SAG1 antibody as per the protocol for indirect immunofluorescence described above. A set of criteria were established to quantify trails. For trails that do not have a parasite associated with them, it is often difficult to visualize the continuity of the trail. For this reason, only trails with a tachyzoite associated with the end were counted. A trail was considered circular if the diameter was 11 μm or less, a cut-off that was determined by measuring and averaging the diameters of 66 circular trails, obtaining the standard deviation (SD), and using the upper limit of the mean plus SD; trails that were larger in diameter or straight were counted as non-circular. Between 25 and 70 trails were enumerated per strain/treatment in each experiment (*n* = 4). For cytoskeleton conformation quantification, parasites were stained with rabbit anti-SAG1 and mouse anti-alpha tubulin, and counted in a blind fashion, i.e., the identity of the samples was not known by the enumerator. At least 85 parasites were counted for each sample. Parasites whose microtubules crossed or overlapped were categorized as helical, whereas those that were aligned and projected in the same direction were counted as straight.

### Live videomicroscopy gliding assay.

1 T25 flask of parasites was filter-purified, centrifuged, and resuspended in 5 ml of HHE. 1 ml of parasites was placed in a tissue culture dish and spun at 1,000 rpm for 5 min to settle parasites onto the dish. Dishes were then placed onto a 37 °C heating block for 2 min to activate parasites, and quickly transferred to the 37 °C heated chamber of an inverted microscope. Videos were captured at 1 frame per s for 1 min using Simple PCI software and the type of gliding/movement was enumerated by observing individual parasites. At least 430 parasites were observed and enumerated for each strain. Maximum projections showing motility patterns over a 1 min period were also generated using Simple PCI software.

### Induced egress assay.

A23187 induced egress was performed as previously described [15]. Briefly, subconfluent HFF monolayers grown on glass well dishes were inoculated with tachyzoites and allowed to grow for 40 h in the presence or absence of ATc. The media in the dishes was changed from D10 Complete to HBSSc (Hank's balanced salt solution, 1 mM MgCl_2_, 1 mM CaCl_2_, 10 mM NaHCO_3_, 20 mM HEPES). The dishes were placed on the 37 °C heated stage of an inverted microscope, and A23187 was added to a final concentration of 5 μM. Real-time egress was captured using Simple PCI software at 2 frames per s and converted into videos played at 5X normal speed. For DTT-induced egress, the protocol of Stommel et al. [16] was followed with the following modifications: the media of the dishes was replaced with fresh D10, and DTT was added to a final concentration of 5 mM.

### In vivo virulence assay.

Groups of female BALB/c mice were infected intraperitoneally with 5 × 10^4^ tachyzoites of tTA-dhfr, *mic2e/mic2i*, or *Δmic2e/mic2i* mutant parasites. In treatment groups, the drinking water was supplemented with 0.2 mg of ATc/ml. The survival of mice after infection was monitored over a period of 6 wk, and seroconversion of all surviving mice was confirmed by ELISA analysis 3 wk after infection. Results of three independent experiments are presented. Care and handling of animals was in accordance with institutional guidelines and approved by the Johns Hopkins University Institutional Animal Care and Use Committee.

### Parasite tissue burden and rtqPCR.

Liver, spleen, heart, lungs, and brain were removed, weighed, and homogenized in PBS; peritoneal flushes were centrifuged and the pellet used for DNA extraction. 2.5% or 5% of the homogenate was used for genomic DNA isolation using the Qiagen (Valencia, California, United States) DNEasy extraction kit. 10% of this DNA was used in each rtqPCR reaction using the SYBR green master mix (PE Applied Biosystems, Foster City, California, United States) and Tox-9 and Tox-11 primers [27]. Numbers of parasites per mg of tissue were calculated using a standard curve generated using purified RH tachyzoites. Cytokine levels were determined by a quantitative cytokine protein microarray analysis by Allied Biotech Incorporated (Ijamsville, Maryland, United States).

### Statistical analyses.

Prism GraphPad software was used for statistical analysis. Two-tailed Student's *t*-tests were used for analysis of invasion, attachment, and gliding assays. A *p*-value of less than 0.05 was considered significant for tests. For correlation analysis, a two-tailed Pearson test and a Spearman test were used.

## Supporting Information

Figure S1 Confirmation of *mic2i* and the Absence of *mic2e* in Clones by PCR and Southern Blot (931 KB JPG)

Figure S2 MIC2 Does Not Influence Intracellular Replication (773 KB JPG)

Video S1 Time-Lapse Video at 5X Speed of tTA-dhfr Parasites after Induced Egress (7.9 MB AVI)

Video S2 Time-Lapse of Δ*mic2e/mic2i* + ATc Induced Egress (8.0 MB AVI)

Video S3 Live Gliding Video at 5X Speed of tTA-dhfr Parasites (9.2 MB AVI)

Video S4 Video of Gliding Δ*mic2e/mic2i* + ATc Parasites (11 MB AVI)

### Accession Numbers

The GenBank (www.ncbi.nlm.nih.gov/Genbank/index.html) accession numbers for the proteins discussed in this paper are AMA1 (AF010264), MIC2 (U62660), MIC3 (AJ132530), MIC4 (AF143487), MIC5 (Y09782), MIC6 (AF110270), MIC10 (AF293654), and M2AP (AF364813).

## Abbreviations

ATc: anhydrotetracycline
CAT: chloramphenicol acetyltransferase
dhfr-ts: dihydrofolate reductase-thymidylate synthase
MIC: micronemal protein
PV: parasitophorous vacuole
rtqPCR: real-time quantitative PCR
tTA: transcriptional transactivator protein
